# Individual dietary specialization reduces intraspecific competition, rather than feeding activity, in black amur bream (*Megalobrama terminalis*)

**DOI:** 10.1038/s41598-020-74997-8

**Published:** 2020-10-21

**Authors:** Yuguo Xia, Yuefei Li, Shuli Zhu, Jie Li, Shanghao Li, Xinhui Li

**Affiliations:** 1 Pearl River Fisheries Research Institute, Chinese Academy of Fishery Sciences, Guangzhou, 510380 China; 2 Experimental Station for Scientific Observation on Fishery Resources and Environment in the Middle and Lower Reaches of the Pearl River, Ministry of Agriculture and Rural Affairs, Zhaoqing, 526100 China

**Keywords:** Freshwater ecology, Stable isotope analysis

## Abstract

Individual specialization and high plasticity in feeding activity are common in natural populations. However, the role of these two in intraspecific competition is unclear. In this study, the rhythm of feeding activity, dietary composition, niche width, niche overlap, and individual specialization was explored in four different size groups of black amur bream (*Megalobrama terminalis*), using microscopic identification of foregut contents and stable isotope analysis (*δ*^13^C and *δ*^15^N) of dorsal muscle. Both methods observed ontogenetic shifts in dietary preference and individual specializations, and revealed that the total niche width of large individuals was greater than small individuals. Mixed linear models indicated that feeding activity was significantly influenced by time (*p* < 0.0001), and no significant changes among size groups was evident (*p* = 0.244). Niche overlaps revealed that there was intensive diet competition between different size groups of black amur bream. Individual specialization in small juveniles was likely to be stronger than sub-adult and adult groups. Pearson’s correlation analysis revealed that the individual specialization was positively correlated with mean diet similarity within a group. The results indicated that intraspecific competition is reduced mainly by individual dietary specialization, rather than shift in feeding activity.

## Introduction

Competitive processes in population ecology influence the spatial and temporal patterns in the members of a population, and how this works has always been a great concern for ecologists^1^. In a given ecosystem, competition refers to the symbiotic interactions between organisms for limited resources, including interspecific and intraspecific competition, and this limits population densities^2^. Intraspecific competition is generally more intense than interspecific competition due to the same requirements for reproduction and growth^3^. Competitions for food, living space, and a mate (or a combination of these) were considered as the main types of intraspecific competition. Food competition could influence fish behavior and offspring survival in the short term, and reduce fish growth and delay maturing in the long term^4,5^. Low abundance of available food resources promotes diversification of fish by size, indicating that body size distribution is affected by competitive interactions^6,7^. Individuals with a similar body size within the same species present more intense competition than those with large size differences^1^. Current data suggest that trophic polymorphism and behavioral differences are important to reduce intraspecific competition in fish species^8^. Behavior patterns in fishes are often said to be plastic. In a laboratory experiment, time of food availability has a great effect on goldfish (*Carassius auratus*) activity, and different individuals present different activity patterns^9^.

Individual specialization is defined as an individual using a small subset of the population’s resource base, and can widely be found in a variety of animals, and even in individuals within a sex and age class^10,11^. Empirical studies have shown that individual specialization is found in many ecological attributes, such as fecundity, resource use, and susceptibility to predation, which is attributed to genetic and environment-based variation^12–14^. Between-individual differences in competition, predation, and pathogen infection risk could be caused by niche divergence^15,16^, thus it is important to understand the strength of individual specializations within species, and variations among populations for evaluation of population dynamics. Individual specialization in diet plays a vital role in intraspecific variation, such as intestine length, and jaw structure^17^. Intra- and interspecific competition, diversity of available resources, and predation may modify the strength of individual specialization^11^.

Optimal foraging theory (OFT) hypothesizes that the animal’s diet will be such that the net rate of energy intake is maximized, depending on individuals’ capacity to capture and digest those resources^18^. OFT explains why dietary differences between individuals of a population occur. Changes in consumer choice occur as the number of competing individuals increases^19^. The range of food items of a species increases as the number of individuals increases within a population, and the extent of individual specialization will also increase^20^. The most competitive or dominant individuals in a population negatively affect the foraging efficiency of weaker conspecifics, that then lose the best foraging areas, and have reduced access to preferred resources^21–23^. This demonstrates that intraspecific competition strengthens individual specialization.

In the present study, the role of feeding rhythm divergence, and individual specialization in reducing intraspecific dietary competition in a freshwater ecosystem was explored. Black amur bream (*Megalobrama terminalis*) is an economically important, omnivorous species which is abundant in the lower reaches of the Pearl River in China. It has been reported that black amur bream mainly consume golden mussel (*Limnoperna fortunei*), Asian clam (*Corbicula fuminea*), organic debris, and aquatic plants^24^. Small subunit ribosomal DNA sequencing analysis revealed that the black amur bream presented dietary shifts during gonad maturation^25^. During the past decade, biomass percentage of black amur bream in catches has decreased by 18.3%^26,27^, and the stock status of this species is overexploited^28^. Therefore, it is important to assess the implications of population niche, niche overlap, and variation in individual specialization of black amur bream in fish stock decline.

## Results

### Gut content composition

A total of 294 black amur bream were collected; the size of specimen ranged from 90 to 345 mm TL with a mean size (± SD) of 212 ± 50 mm. All samples were divided into four size groups based on standard length and gonadosomatic index. Among them 65 (22%), 91 (31%), 96 (33%), and 42 (14%) individuals were small juvenile, large juvenile, sub-adult and adult, respectively (Table 1). A total of 28 prey items from 37 intestinal tracts were assigned to taxa. The prey items that were identified to the order level represent over 98% (%W and %N) of all prey examined in each group (Table 2). The percent by weight of gut contents was dominated by detritus across all groups with an average biomass percentage of > 84%. Small juveniles consumed a wider variety of prey that consisted of 20 prey taxa. Besides detritus, the most common prey taxa were Chaetophorales (5.5%W), followed by Coscinodiscales (0.2%W), and Araphidiales (0.2%W). Large juveniles fed on three major prey taxa besides detritus, Mytiloida, Chaetophorales and Ulvales, with biomass percentages of 13.8%, 1.4% and 0.1%, respectively. Sub-adult individuals mainly fed on detritus and Mytiloida, comprising 96.8% and 3.1% by weight respectively. Detritus and Mytiloida also dominated the adult diet, comprising 92.7% and 7.2% by weight, respectively.

**Table 1:** Basin biological information of different size groups of black amur bream collected in the Pearl River, China.

Group	SL (mean ± SD)	Wt (mean ± SD)	GSI (mean ± SD)	N
Small juvenile	143.6 ± 17.5^a^	66.8 ± 26.9^a^	0.27 ± 0.31^a^	65
Large juvenile	193.9 ± 14.6^b^	163.2 ± 43.6^b^	0.73 ± 1.03^a^	91
Sub-adult	241.5 ± 14.4^c^	323.8 ± 84.8^c^	2.06 ± 2.51^b^	96
Adult	287.3 ± 18.5^d^	566.2 ± 165.0^d^	4.5 ± 3.51^c^	42

Different superscript letters indicate significant differences (Tukey HSD, *p* < 0.05). N, sample size, and same as follows.

**Table 2:** Identifiable black amur bream prey in each size group sorted by taxa. Items with a percentage by weight over 0.1% and percentage by number over 1% are listed.

Food item	Small juvenile (N = 9)	Large juvenile (N = 6)	Sub-adult (N = 15)	Adult (N = 7)
**Percent by weight % (%W, mean ± SD)**				
Detritus	94.1 ± 16.4	84.5 ± 33.3	96.8 ± 7.0	92.7 ± 14.8
Chaetophorales	5.5 ± 16.4	1.4 ± 3.0	0	0
Mytiloida	0	13.8 ± 33.9	3.1 ± 7.1	7.2 ± 14.9
Coscinodiscales	0.2 ± 0.2	0.02 ± 0.04	0.08 ± 0.15	0.05 ± 0.08
Araphidiales	0.2 ± 0.5	0.05 ± 0.12	0.04 ± 0.08	0.01 ± 0.01
Ulvales	0	0.1 ± 0.3	0	0
**Percent by number % (%N, mean ± SD)**				
Coscinodiscales	53.6 ± 39.3	9.4 ± 22.6	45.0 ± 32.6	50.2 ± 36.7
Chaetophorales	12.5 ± 35.3	43.4 ± 49.1	0	0
Chlorococcales	11.7 ± 26.0	0.1 ± 0.3	16.0 ± 29.7	14.4 ± 21.1
Mytiloida	0	16.7 ± 40.8	0.02 ± 0.03	0.02 ± 0.04
Araphidiales	5.9 ± 12.6	2.4 ± 5.8	11.7 ± 11.2	6.7 ± 7.9
Chroococcales	0.1 ± 0.3	0	0	26.2 ± 38.7
Osillatoriales	1.0 ± 2.9	0	9.9 ± 17.5	0
Ulvales	0	6.1 ± 13.5	0	0
Biraphidinales	4.4 ± 6.5	21.8 ± 36.0	15.5 ± 30.3	1.1 ± 1.7
Aulonoraphidinales	9.3 ± 20.5	0	0.5 ± 1.5	0.4 ± 0.6

Percentage by number excludes detritus.

The percent by number of prey taxa in a given individual gut was different to the percent by weight due to the different volumes. The amount of detritus was hard to determine, and we excluded while calculating the percent by number. Four prey taxa, Coscinodiscales, Chaetophorales, Chlorococcales and Aulonoraphidinales dominated the small juvenile diet, cumulatively comprising 87.1% by number (Table 2). The prey taxa, Chaetophorales, dominated the large juvenile diet, comprising 43.4% by number. Sub-adults mainly fed on Coscinodiscales comprising 45.0% by number, and Aulonoraphidinales and Mytiloida were also recorded in rare instances (Table 2). The most abundant prey taxon was Coscinodiscales (50.2%N) in the adult group, and Araphidiales, Biraphidinales, Aulonoraphidinales, and Mytiloida were also found in some adult individuals.

Overall, the gut content compositions of the different size groups did not vary greatly, as indicated by small variations for the major prey taxon detritus in percent by weight (Table 2). However, the prey taxa varied greatly between size groups in percent number. Black amur bream demonstrated different diet preferences in relation to body length.

### Feeding activity

**Figure 1:**
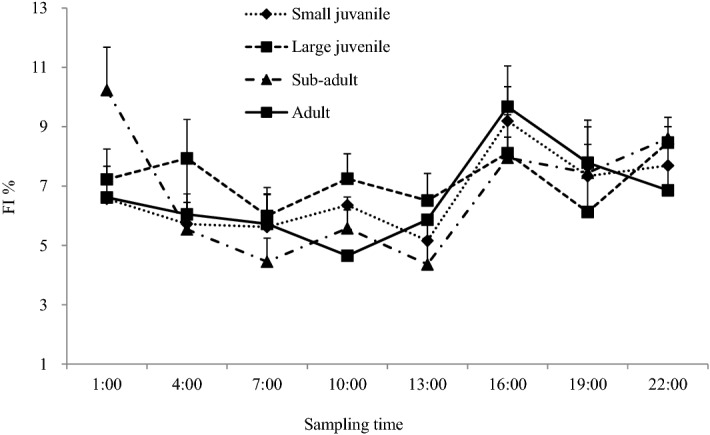
Variation in the gut fullness index (%FI, mean ± SE) for the four size groups of black amur bream analyzed during a 24 h sampling period. This figure created using EXCEL version 2010.

During the sampling period of 24 h, the black amur bream was considered to be actively feeding at all times (Fig. 1). The highest fullness index (FI) of small juvenile was observed around 16:00, and the lowest around 13:00. After sunset, before mid-night (19:00–22:00), the gut fullness stayed at an intermediate level. From 1:00 to 10:00, the gut fullness was at a low level. The large juveniles generally had a greater fullness than small juveniles, and feeding activity did not vary across all times. The highest gut fullness in sub-adults was observed around 1:00, and sharply decreased by 4:00, and continued to decline until 7:00. During the late morning, (7:00–13:00), gut fullness was observed to be low. From 16:00 to 22:00, the gut fullness slightly increased and stayed at a medium level. Mean gut fullness of adult individuals was low during the early morning (1:00–7:00), and declined to a minimum at 10:00. During the afternoon, the gut fullness greatly increased and peaked at 16:00, followed by slight decline after sunset (19:00–22:00).

The FF was the response of the mixed linear model; size group, sampling time and the interaction of these two factors were fixed effects; sampling season was the random effect. The results of the ANOVA tested models indicated that sampling time (χ^2^ = 21.432, *p* < 0.0001) was a significant influence on the feeding activity. The size group did not significantly affect the fish feeding activity (χ^2^ = 1.358, *p* = 0.244). This demonstrated that there was no significant change in feeding rhythm among individuals.

### Trophic niche widths

**Figure 2:**
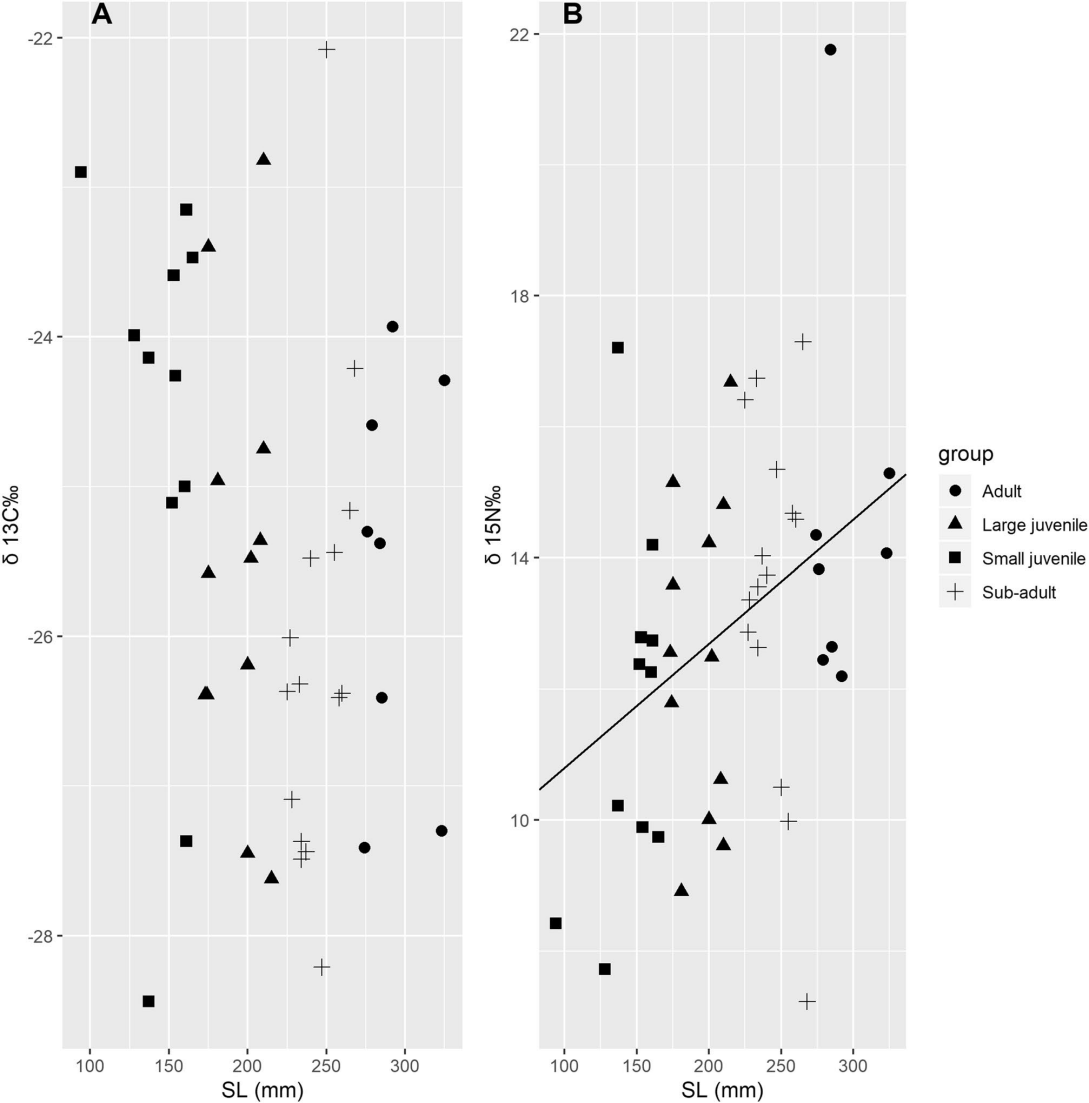
The relationship between isotope values and standard length of black amur bream. This figure created using R software version 3.6.1 (https://www.r-project.org/).

A total of 46 samples of four size groups of black amur bream and 40 samples of potential prey sources were analyzed for *δ*^13^C and *δ*^15^N. There was a high degree of isotope overlap in *δ*^13^C and *δ*^15^N between the size groups (Table 3). The values for black amur bream ranged from − 28.44 to − 22.08‰ for *δ*^13^C and from 7.23 to 21.76 ‰ for *δ*^15^N. Curve estimation indicated that the *δ*^15^N value of the black amur bream is a linear function of standard length. All coefficients were statistically significant, and the R-squared value was approximately 0.133 (Fig. 2B, *δ*^15^N = 8.90 + 0.02 × SL, *p* = 0.013). The *δ*^13^C value was negatively correlated (linearly) with fish standard length, but the relationship was not significant (*p* = 0.134, Fig. 2A). The *δ*^13^C and *δ*^15^N of potential prey taxa ranged from − 31.00 to − 13.07‰, and from 2.87 to 17.35‰, respectively. Riparian C_4_ plants had the highest *δ*^13^C and lowest *δ*^15^N values, with mean values of − 13.29 ± 0.21 ‰ and 4.06 ± 1.44 ‰, respectively. The lowest *δ*^13^C and highest *δ*^15^N values were distributed in *Corbicula fluminea* and *Macrobrachium nipponense*, respectively. The other prey taxa had a wider variety of *δ*^13^C and *δ*^15^N values, but overlapped with each other to some extent. The mean values and standard deviations for each fish group and prey taxon for *δ*^13^C and *δ*^15^N are shown in Table 3. The C/N ratio (% weight) in potential prey taxa ranged from 3.17 to 77.32, where *M*. *nipponense* had the minimum value, and riparian C_4_ plants had the maximum value.

**Table 3:** Summary statistics (mean ± SE) of *δ*^13^C, *δ*^15^N, and C/N in the different size groups of black amur bream and potential prey sources in the sampling site.

Group/taxon	Code	N	*δ* ^13^C (‰)	*δ* ^15^N (‰)	C/N
Small juvenile	Small juvenile	11	− 24.67 ± 1.75	11.60 ± 2.75	–
Large juvenile	Large juvenile	12	− 25.53 ± 1.43	12.54 ± 2.44	–
Sub-adult	Sub-adult	15	− 26.10 ± 1.52	13.53 ± 2.69	–
Adult	Adult	8	− 25.58 ± 1.33	14.57 ± 3.09	–
Zooplankton	Zooplankton	2	− 30.25 ± 1.07	9.95 ± 2.08	6.11 ± 0.66
Phytoplankton	Phytoplankton	2	− 27.94 ± 0.24	8.21 ± 1.13	8.40 ± 1.86
Riparian C_4_ plants	C_4__P	4	− 13.29 ± 0.21	4.06 ± 1.44	63.89 ± 10.35
*Potamogeton* sp.	Psp	6	− 25.29 ± 3.26	7.45 ± 2.43	10.46 ± 1.26
*Macrobrachium nipponense*	Mni	9	− 26.97 ± 0.66	15.95 ± 1.32	3.30 ± 0.07
*Anodonta woodiana*	Awo	2	− 24.72 ± 0.65	6.46 ± 0.04	3.84 ± 0.06
*Limnoperna fortunei*	Lfo	1	− 26.53	4.96	4.65
*Semisulcospira cancellata*	Sca	1	− 24.16	9.51	3.91
*Corbicula fluminea*	Cfl	4	− 30.27 ± 0.26	11.99 ± 0.31	4.65 ± 0.43
*Bellamya* sp.	Bsp	4	− 22.28 ± 0.34	4.42 ± 1.02	4.14 ± 0.27
Benthic detritus	Bde	2	− 26.11 ± 1.20	7.08 ± 1.50	12.57 ± 4.50
Sediment	Sediment	3	− 25.23 ± 0.39	5.85 ± 0.23	10.47 ± 1.23

Values are mean ± SD.

The mean proportional contribution of potential prey sources to black amur bream varied among taxa, observing a range from 0.02 to 0.15 (Table 4). The contribution of 12 potential taxa to small juveniles ranged from 0.05 to 0.10, with *C. fluminea* in the highest proportion, and C_4__P in the lowest proportion. The mean contributions of other prey to small juveniles were almost similar. The riparian C4 plant contributions were always low across all size groups. The mean proportional contributions of zooplankton, *M. nipponense* and *C. fluminea* were high, demonstrated to be very important for black amur bream. The contributions of *M. nipponense* and *C. fluminea* increased from small to adult individuals, and contributions of prey sources within groups also increased with body length. This appeared to be a dietary shift during fish growth.

**Table 4:** Contributions of different potential prey taxa to different size groups of black amur bream, according to stable isotope Bayesian mixed models.

Prey	Small juvenile		Large juvenile		Sub-adult		Adult	
	Mean	CI95%	Mean	CI95%	Mean	CI95%	Mean	CI95%
Zooplankton	0.09	0–0.18	0.11	0–0.21	0.13	0–0.25	0.11	0–0.21
Phytoplankton	0.08	0–0.17	0.09	0–0.19	0.09	0–0.20	0.09	0–0.19
C_4__P	0.05	0–0.11	0.03	0–0.07	0.02	0–0.06	0.03	0–0.08
Psp	0.09	0–0.17	0.08	0.01–0.18	0.07	0–0.18	0.08	0–0.19
Mni	0.09	0–0.17	0.11	0–0.20	0.14	0.03–0.25	0.15	0.02–0.27
Awo	0.08	0–0.17	0.07	0–0.17	0.05	0–0.14	0.06	0–0.15
Lfo	0.09	0–0.17	0.08	0–0.17	0.06	0–0.15	0.06	0–0.16
Sca	0.08	0–0.17	0.07	0–0.17	0.06	0–0.15	0.07	0–0.16
Cfl	0.10	0–0.18	0.13	0.01–0.23	0.15	0.01–0.29	0.12	0.01–0.23
Bsp	0.08	0–0.16	0.06	0–0.14	0.04	0–0.10	0.05	0–0.13
Bde	0.08	0–0.17	0.08	0–0.18	0.09	0–0.20	0.09	0–0.19
Sediment	0.08	0–0.17	0.08	0–0.18	0.09	0–0.20	0.09	0–0.19

CI95%, lower—higher confidence intervals.

The total trophic niche width was assessed using both gut contents and stable isotopes through TNW and SEA_c_, respectively. The higher values indicated a greater niche width in a given group. Both methods showed consistent results, with the trophic niche width increasing with body length. The values of the TNW and SEA_*c*_ varied among size groups: adult > sub-adult > large juvenile > small juvenile (Table 5).

**Table 5:** Metrics quantifying trophic niche and individual specialization in black amur bream.

	Small juvenile	Large juvenile	Sub-adult	Adult
Taxonomic richness per gut (mean ± SD)	5.5 ± 3.0^a^	2.8 ± 1.5^a^	4.8 ± 2.2^a^	4.8 ± 1.6^a^
TNW	0.058	0.699	0.862	0.938
WIC/TNW	0.250	0.951	0.637	0.610
Diet similarity	0.339	0.185	0.375	0.386
NR (‰)	9.49	7.77	10.07	9.56
CR (‰)	5.54	4.80	6.13	3.48
CD (‰)	2.61	2.40	2.47	2.55
MNND ± SD(‰)	1.04 ± 1.07	1.37 ± 0.62	1.15 ± 1.10	1.69 ± 2.05
TA	19.22	21.74	30.87	17.52
SEA (‰)	9.446	10.041	11.240	12.957
SEA_c_ (‰)	10.496	11.045	12.104	15.116

Same superscript letters indicate no significant differences (Kruskal–Wallis test, *p* > 0.05).

### Niche overlap

**Figure 3:**
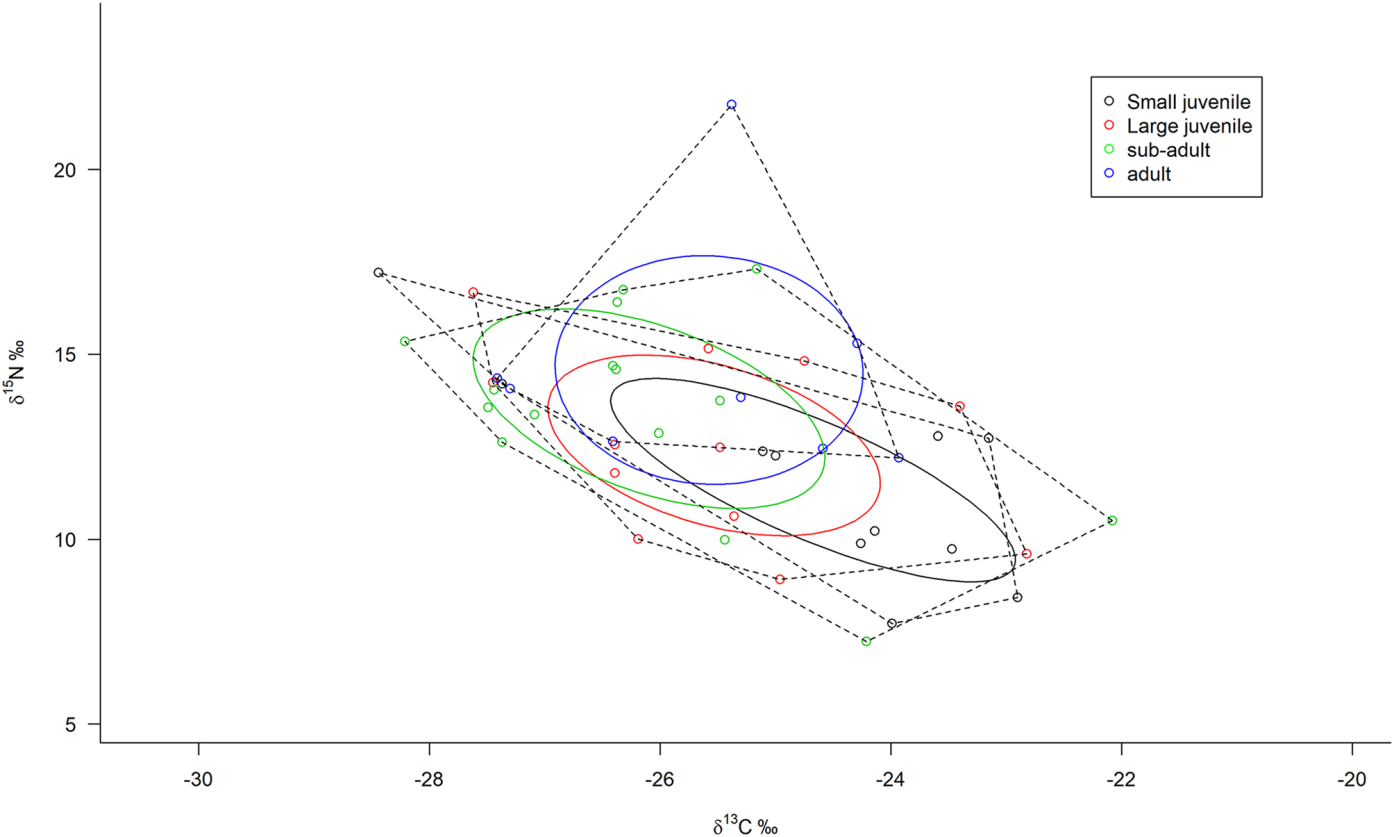
The area of convex hulls (TA) of*δ*^13^C and *δ*^15^N values plotted by dashed lines according to the four size groups of black amur bream. The area of standard ellipse (SEA_*c*_) is drawn by solid line, and contains 40% of the data. This figure created using R software version 3.6.1 (https://www.r-project.org/).

The pairwise Morisita’s dietary overlap index was generally > 0.6 between any two size groups, and very close to 1. Both carbon and nitrogen isotopic values exhibited no significant difference between the four size groups (ANOVA, *p* = 0.156 and 0.104 for *δ* ^13^C and *δ* ^15^N respectively). These similarities suggested niche partitioning among the four size groups, with large SEA_*c*_ overlap (Fig. 3). The SEA_*c*_ of four size groups overlapped with each other on the isotope biplot, with the largest overlap area occurring between sub-adult and adult, whereas the smallest overlap occurred between small juveniles and adult (Table 6). Both methods (Morisita’s index and SEA_*c*_ overlapping) indicated a high potential for resource competition within the population of black amur bream.

**Table 6:** Pairwise trophic overlap using Morisita’s index and geometric overlap area of corrected standard ellipse area (SEA_*c*_) among four size groups of black amur bream.

	Small juvenile	Large juvenile	Sub-adult
**Morisita’s dietary overlap index**			
Large juvenile	0.982		
Sub-adult	0.997	0.984	
Adult	0.995	0.993	0.998
**SEA**_***c***_** overlap (‰**^**2**^**) between groups (percentage in parentheses)**			
Large juvenile	45.96 (0.52)		
Sub-adult	46.62 (0.48)	51.29 (0.59)	
Adult	44.23 (0.39)	51.81 (0.51)	54.18 (0.53)

### Individual specialization

Individual prey specialization was assessed using both gut contents and stable isotopes through the WIC/TNW_g_ and CD, respectively. Low WIC/TNW_g_ and high CD both indicated less similarity between individuals. Both methods provided higher individual specialization in small juveniles than in sub- and adult groups. Pearson’s correlation analysis revealed that mean pairwise diet similarity was negatively correlated with WIC/TNW_g_ (Pearson’s correlation = − 0.642, *p* = 0.358).

The NR was found to be larger for sub-adults and adults than for juvenile groups, whereas CR was high in the sub-adult group, and low in the adult group (Table 5). A slightly higher mean trophic diversity (assessed by CD) was found in small juveniles compared with other size groups. The RPP test indicated that the CD in the small juveniles did differ significantly from the sub-adult (*p* = 0.026) and adult (*p* = 0.019) groups. The MNND did not differ significantly between size groups (*p* > 0.05). The Hotelling’s *T*^2^ tests revealed that centroid location did not differed significantly (Euclidean distance between centroids was not significantly different from zero) for the contrast of the small juveniles and adults (Hotelling’s *T*^2^ = 5.81, *p* = 0.089).

## Discussion

Although black amur bream have been an economically important species in the Pearl River basin for many decades, only a handful of studies have explored their feeding ecology^24,25^. The goal of the present study was to improve the understanding of intraspecific dietary competition, and its influencing factors, which is important to the protection and sustainable use of black amur bream. Gut contents and stable isotope analyses were both used to compare feeding rhythm, niche overlap, and individual specialization among four size groups.

### Prey composition different revealed by gut content and stable isotope

Remains of prey in the foregut samples were difficult to identify by microscopic observation due to digestion. The proportions of prey items in black amur bream were very different by weight or by number. Dietary preference shifts were observed during fish growth, where juveniles preferred to consume phytoplankton and adult consumed more animal material. Dietary shift is common in fish and are closely related to the ontogenetic changes in energy demands, and foraging performance^29,30^. Dietary composition of the black amur bream is supported by a previous study in the Pearl River with a high consumption of detritus^24^. Small subunit ribosomal DNA (18S rDNA) sequencing also detected juvenile black amur bream with highly abundant Streptophyta, and adults with more benthic animals^25^. The 18S rDNA sequencing method identified prey items along with intestinal microbes, while gut content identification can more accurately determine the contribution of each item. However, microscopic evaluation of foregut contents overestimated detritus, but this did not great affect the calculation of dietary overlap due to the high percentage of detritus in each size group.

Stable isotope analysis provides a powerful tool to characterize carbon sources and trophic position^31^, but is biased to detecting specific trophic interactions as isotope values in potential prey often overlap^32^. The stable isotope analysis showed that almost all prey taxa contribute to black amur bream nutrition (Table 4), which may be attributed to the trophic interactions among prey items. However, isotope analysis revealed the variation in the contribution of given prey items to the four size groups with results concurring with gut content analysis. The number of prey items determined by isotope analysis was much less than visual, and molecular identification of gut contents^25^. Two isotope ratios powerful evaluated the flow of organic material from three prey items to consumers^33^, but it is hard to determine more than three preys due to the variation in trophic fractionations between preys and consumers^34–36^.

### High intraspecific dietary competition of black amur bream

In addition to the shift in dietary preference, there is an increase in the niche width of black amur bream during fish growth. Competing theories assumed that ecosystem productivity increases the niche width of omnivores, which decreases as consumers utilize distinctive prey items^37,38^. Trophic niche width is also closely related to the intensity of competition, which tended to increase under some environmental conditions^39^. Large individuals are generally superior to small sized individuals when competing for food, with a higher growth rate and wider dietary sources^40^. The niche width of adult black amur bream was larger than juvenile individuals may mainly due to ontogenetic changes in feeding ability.

Both methods visual identification and isotope analysis consistently revealed high intraspecific dietary competition in black amur bream. It has been reported that the primary production of phytoplankton in the mainstream of the Pearl River is less than 400 mg (m^2^ d)^−1^, and biomass of riparian and submersed plants is very low^41,42^. Low abundance of available prey resources could be the reason for high intraspecific competition, and busy feeding activities occurring during the 24 h sampling period seem to prove this hypothesis (Fig. 1). The proportion of starving black amur bream (FF = 0 or 1) was about 11% by number.

### Individual specialization reduces intraspecific dietary competition

Variation among individuals within populations is an evidence of competition, and has important consequences for ecological interactions^13^. Natural selection holds that competition intensity should decline over time among sympatric populations due to adaptive change, facilitating natural population persistence^43,44^. Generally, high individual specialization represents populations that possess considerable diversity and great variation, promoting resource portioning and coexistence. The highest individual specialization was found in small juvenile black amur bream (lowest WIC/TNW), which may be explained by the high individual variation cause by limited foraging abilities within this group. Diet similarity between individuals in a group was negatively correlated with WIC/TNW, which demonstrated that intraspecific diet competition was positively correlated with individual specialization. On average, the WIC/TNW value was 0.66 ± 0.21 (n = 78 populations), and individuals’ niches were narrower than population niches^11^. The value of WIC/TNW_g_ in small juvenile black amur bream was much less than the average level, indicating high intraspecific competition. Low foraging efficiency and high conspecific densities may contribute to high competition in the small juvenile group^45,46^. Low variation among individuals in sub-adults and adult may be due to exploitation competition, where one individual depletes a food patch before another one arrives^4^. An extremely high value of WIC/TNW_g_ was found in the large juvenile group (Table 5), probably because of the low prey taxonomic richness, which may underestimate the dietary variations among individuals within this group. The CD demonstrated same variation patterns with WIC/TNW_g_. However, CD in the small juveniles did not significantly differ from the large juveniles (*p* = 0.330), which proves that WIC/TNW underestimates the dietary variations in the large juvenile group.

## Conclusions

Ontogenetic changes in dietary preference and plasticity in feeding activity are common in fish; however, the role of these two in intraspecific competition is unclear. The results showed that juvenile black amur bream preferred to consume phytoplankton and adult consumed more animal material. However, there were no ontogenetic changes in rhythms of feeding activity of black amur bream. Although dietary shift was found in black amur bream, there was great dietary overlap and intense competition between different sized individuals. Moreover, diet similarity within a size group was positively correlated with individual specialization. The results indicated that intraspecific diet competition was reduced by individual specialization, rather than divergence in feeding activity. The challenge for the future is to understand the contribution of genetic variance and morphological feeding differences to individual specialization, and how the strength of individual specialization changes with ecological interactions.

## Materials and methods

### Sample collection and preparation

The Pearl River is the longest river in southern China, with a total length and annual discharge of 2214 km and 10,000 m^3^s^−1^, respectively. The present study was conducted in the Zhaoqing section (23°02ʹ–04ʹ N, 112°25ʹ–31ʹ E) in the lower reaches of the Pearl River. Fish samples were collected in the dry season of 2015–2017, and the flood season of 2017 and 2019. Sampling was carried out with circular cast nets (15 m diameter, mesh size 4 cm). Eight daily hauls of 1 h each were performed every 3 h throughout a 24 h period to cover the entire diel cycle. The samples, kept on ice immediately and subsequently refrigerated, were transported to the laboratory. Each fish was measured for standard length (SL) to the nearest 1 mm, weighed for body weight (W_t_), gonad weight (W_g_) and gut weight (W_gut_) to the nearest 0.1 g, and sex and maturity stage was recorded. The foregut was removed, and the contents were weighed to the nearest 0.0001 g. A sample of dorsal muscle was dissected and stored at − 20 °C for stable isotope analysis. Gonadosomatic index (GSI) and fullness index (FI) were calculated by the biomass percentage of gonad and gut to body mass, respectively. The GSI and FI were defined as follows:

$${\text{GSI }} = { 1}00 \times {\text{W}}_{{\text{g}}} /{\text{W}}_{{\text{t}}} ,$$

$${\text{FI }} = { 1}00 \times {\text{W}}_{{{\text{gut}}}} /{\text{W}}_{{\text{t}}} .$$

Potential prey sources including phytoplankton, zooplankton, invertebrate, riparian C_4_ plants, submersed plants, benthic detritus and sediment were collected in the dry season of 2017 and 2018. Phytoplankton was collected by a horizontally hauled phytoplankton net (25#, 64 μm) from 0.5 m below the surface, and repeated until enough sample was collected. The phytoplankton samples were suspended in distilled water for at least 2 h, and then passed through plankton net (13#, 112 μm) to remove zooplankton and large particles. The phytoplankton was filtered onto pre-combusted Whatman GF/F glass fiber filters (0.7 μm, 450 °C for 8 h). Zooplankton samples were collected using the plankton net (13#), with the same preparation method as phytoplankton.

Benthic macroinvertebrates were collected with a weighted Petersen grab (1/16 m^2^), and the samples were sieved through a 420 μm sieve^47^. In the laboratory, benthic animals were picked and kept in distilled water for 12 h to empty their gut contents. The benthic detritus was collected from the residue after the benthic animals were removed. Sediment samples were collected using Petersen grab, benthic animals were removed, and the sample was divided into two parts at each sampling site. One part was acidified with hydrochloric acid vapor in an enclosed space for 24 h to remove any carbonate for carbon isotope analysis, and the other part used for nitrogen isotope analysis. Plants were collected by hand, and then rinsed in distilled water. Riparian C_4_ plants were dominated by *Hemarthria altissima* and *Cynodon dactylon*, and submersed plants included *Potamogeton crispus* and *P*. *pectinatus*. Several individuals (5–20) of small species like *Corbicula fluminea* and *Limnoperna fortune* were pooled to make a single sample of adequate sample mass. The preparation of macroinvertebrates was as previously described^48^.

All samples for stable isotope analyses were freeze-dried at < 40 Pa and < − 40 °C for 48 h (FD-1-50 Plus, BIOCOOL) and then ground into a fine powder using an automated vibration ball grinder (except for the filtered samples). The stable isotopes *δ*^13^C and *δ*^15^N were measured using a Thermo DELTA V Advantage Isotope Ratio Mass Spectrometer with external Flash EA1112 HT Elemental Analyzer equipment. Stable isotope ratios were expressed as parts per thousand (‰) of the international standards. The Vienna Pee Dee Belemnite and atmospheric nitrogen were used as the standards for carbon and nitrogen, respectively. Analytic precision was ± 0.1‰ for δ^13^C and ± 0.2‰ for δ^15^N, respectively. The C% or N% of each sample was calculated using the relative peak area of the sample to a working standard.

### Feeding activity

Fish fullness (FF) was divided into six levels by visual observation of morphology^49^ where 0 is an empty intestine, 1 is 1/4 full intestines, 2 is 1/2 full intestines, 3 is 3/4 full intestines, 4 is full intestines, and 5 is intestinal distension.

The FF was a response variable, and size group and sampling times were fixed effects. A mixed linear model was fitted to analyze the effects of body size and sampling time on feeding activity. Time- and group-level predictors were included in the model (full model: FF ~ Group + Time + Group × Time + (1|Season)), and new models were produced by reducing fixed effects step by step. To test the significance of fixed effects, the full model and other models were compared using ANOVA. Mixed linear models were fitted by the “lme4” package^50^. All statistical analyses were performed using R 3.6.1^51^.

### Trophic niche widths

Foregut contents were identified, and sorted into taxonomic groups down to the order level if possible. Prey importance was expressed by relative numerical abundance (%N), and relative mass abundance (%W). Mean proportional contribution of potential prey sources to black amur bream based on stable isotope ratios was calculated using the Stable Isotope Analysis in R (siar) package^52^. The black amur bream consumed mixtures of plant and animal material, thus the trophic enrichment factor (TEF) was assigned as 1.3 ± 0.3 ‰, and 2.3 ± 0.18 ‰ for *δ*^13^C and *δ*^15^N, respectively^53^.

The mean distance to centroid (CD) measured the Euclidean distance of each sample to the mean *δ*^13^C and *δ*^15^N values, to indicate average degree of trophic diversity. The CD can be partitioned into trophic level diversity (*δ*^15^N range: NR), and carbon matter source diversity (*δ*^13^C range: CR). The isotopic niche width was based on the convex hull area (TA) that was calculated by the smallest convex polygon bounding the individuals in *δ*^13^C and *δ*^15^N niche space^54^. The TA directly measured population niche width, reflecting variation in niche dimensions along two isotopes, is widely used to describe the isotopic niche width of fish^55^. The standard elliptical area (SEA) also represents dietary niche partitioning for each group, and contains 40% of studied individuals of a population without being affected by outliers^56^. The SEA_*c*_ was corrected by sample size in a bivariate distribution, being unbiased with respect to sample size^54^. The mean nearest neighbor distance (MNND) measured the trophic similarity between individuals through Euclidean distances in the biplot space^54^.

TA, SEA, SEA_*c*_ were calculated using the Stable Isotopes Bayesian Ellipses (SIBER) package^54^. The differences between size groups for the centroid location, CD, and MNND were tested using a residual permutation procedure (RPP) and the parametric Hotelling’s *T*^2^ test^57^.

### Niche overlap

Dietary overlap was calculated using the simplified Morisita’s index^58,59^:

$${C}_{ij}=2\left(\sum {p}_{ik}\cdot {p}_{jk}\right){\left(\sum {p}_{ik}^{2}+\sum {p}_{jk}^{2}\right)}^{-1},$$

where *C*_ij_, dietary overlap index for predators *i* and *j*; *p*_ik_ and *p*_jk_, biomass proportions of predators *i* and *j* with prey *k* in their foregut. A *C*_ij_ value over 0.6 is considered a significant overlap.

Isotopic niche overlap was assessed by the extent of the overlapping area between the SEA_c_ of two groups, which was measured by the percentage of overlapping SEA_*c*_ and compared between size groups^60^.

### Individual specialization

The total niche width (TNW) of a population is the variance of total resource use of all individuals, can be divided into two components: the variation within individuals (WIC), and the variance between individuals (BIC)^61^. The relative degree of individual specialization can be assessed by the proportion of TNW explained by WIC, WIC/TNW, and ranges from 0 to 1. Smaller values of WIC/TNW indicate a lower individual overlap, and higher individual specialization^62^. The TNW and WIC of each group was expressed as follows^62,63^:

$$\mathrm{TNW}=-\sum_{k}{q}_{k}ln\left({q}_{k}\right)$$

$$\mathrm{WIC}= \sum_{i}{p}_{i}\left(-\sum_{k}{p}_{ik}ln\left({p}_{ik}\right)\right)$$

where *p*_*i*_ is the proportional numerical abundance of all resources used by individual *i*; *q*_*k*_ is the proportion of the *k*th resource category in a group’s niche, and *p*_*ik*_ is the proportion of resource *k* used by individual *i*. Diet similarity calculates the mean pairwise diet similarity between all individuals in a group. The TNW, WIC/TNW, and diet similarity were calculated for each group by RInSp^64^.

### Ethics statement

All experiments were performed under the approval of the Ethics Committee of Pearl River Fisheries Research Institute, Chinese Academy of Fishery Sciences. All methods were performed in accordance with the Chinese Association for the Laboratory Animal Sciences and the Institutional Animal Care and Use Committee (IACUC) protocols.
